# Secular Trends in Utilization of Critical Care Services Among Candidemia-Associated Hospitalizations: A Population-Based Cohort Study

**DOI:** 10.14740/jocmr2387w

**Published:** 2015-12-03

**Authors:** Lavi Oud

**Affiliations:** Division of Pulmonary and Critical Care Medicine, Department of Internal Medicine, Texas Tech University Health Sciences Center at the Permian Basin, Odessa, TX 79763, USA. Email: lavi.oud@ttuhsc.edu

**Keywords:** Candidemia, Intensive care unit, Epidemiology

## Abstract

**Background:**

The incidence of candidemia continues to rise in recent years. Candidemic patients often require care in an intensive care unit (ICU) and can consume substantial resources. However, there are no contemporary longitudinal population-level data on the incidence patterns of ICU utilization among patients with candidemia in the United States.

**Methods:**

The Texas Inpatient Public Use Data File was used to identify hospitalized patients aged ≥ 18 years for the years 2001 - 2010. Hospitalizations with candidemia were identified by presence of an International Classification of Diseases, Ninth Revision, Clinical Modification Code 112.5. The annual rates of ICU admission among candidemia hospitalizations were examined. The annual incidence of candidemia hospitalizations with admission to ICU (C-ICU) was evaluated using the United States Census data, and was also benchmarked against the number of all hospitalizations and hospitalizations with ICU admission, overall and for age-specific strata.

**Results:**

There were 11,544 hospitalizations with candidemia, including 7,552 (65.4%) with C-ICU. Between 2001 and 2010, the rate of C-ICU among hospitalizations with candidemia increased from 60.2% to 68.0%, and the incidence of C-ICU increased by 91%, rising from 2.73 to 5.21 per 100,000 population. When benchmarked against hospital admissions and ICU admissions, the following changes were noted between 2001 and 2010 in the incidence of C-ICU: 1.61 vs. 3.34 per 10,000 hospitalizations, and 8.33 vs. 13.77 per 10,000 hospitalizations with ICU admission. The incidence of C-ICU rose rapidly during the first half of the last decade, while plateauing during the remainder of study period. There has been marked difference in the rate of rise in the incidence of C-ICU among examined age strata, being highest among the 18 - 44 year group.

**Conclusions:**

ICU care occurred in the majority of candidemia hospitalizations. The incidence of C-ICU rose nearly two-fold during study period, but the rise plateaued during the second half of the last decade. Substantial heterogeneity was noted in the rate of rise in the incidence of C-ICU across examined age groups. Further study in other populations is required to corroborate our findings and examine the sources of the observed trends.

## Introduction

Candidemia has been consistently among most common causes of bloodstream infections (BSI) [1] and has been recently reported as the most common cause of healthcare-associated BSI in a multistate survey in the United States (US) [2]. The incidence of candidemia has been remarkably variable across nations [1, 3] and regionally [4], and was generally [3, 5], though inconsistently [6] showing progressive rise. Although a recent report noted decreasing incidence of candidemia in two metropolitan areas in the US [4], the generalizability of this trend remains unknown.

Candidemia generally develops among patients with chronic comorbid conditions, acute illness, and invasive interventions, often associated with critical illness, being a major cause of sepsis [7] and involving resource-intensive care. Data on the evolving demand for critical care services among candidemic patients can inform plans for critical care resource allocation and future investigations of the drivers of the evolving epidemiological patterns in this population. However, although numerous studies reported on the incidence patterns of candidemia in hospitalized patients [3, 5, 6], there has been a limited number of contemporary longitudinal population-level reports on the incidence of candidemia among patients admitted to an intensive care units (ICU) [8, 9], with none reported in the US.

We sought to examine the evolving general and age-specific incidence patterns of candidemia among adult patients requiring critical care in Texas.

## Methods

We used the Texas Inpatient Public Use Data File (TIPUDF) [10], a publicly available, de-identified dataset maintained by the Texas Department of State Health Services to identify hospitalizations 18 years or older with a diagnosis of candidemia during the years 2001 - 2010. A hospitalization with candidemia was defined as reported International Classification of Diseases, Ninth Revision, Clinical Modification (ICD-9-CM) diagnosis code 112.5 (disseminated candidiasis). Hospitalizations with admission to ICU were defined as presence of an ICU-specific revenue code. US census data of censal reports and intercensal estimates [11] were used to derive data on state population 18 years or older. Because we used a publicly available, de-identified dataset, this study was determined to be exempt from formal review by the Texas Tech Health Sciences Center Institutional Review Board.

We examined the total and age-stratified annual incidence of candidemia hospitalizations with ICU admission (C-ICU) in the general population. In addition, we benchmarked the incidence of candidemia as a function of total annual volume of hospitalizations and those requiring admission to ICU in the state.

Because patterns of the incidence of a given condition managed in the ICU may reflect in part admission rates among all hospitalized patients with the studied condition, we examined the annual admission rate to ICU among all candidemia hospitalizations.

## Results

There were 11,544 hospitalizations with reported diagnosis of candidemia during the years 2001 - 2010. Admission to an ICU was reported in 7,552 (65.4%) hospitalizations. The rate of ICU admission among candidemia hospitalizations increased progressively from 60.2% in 2001 to 68.0% in 2010.

The data on the total and age-stratified annual incidence of C-ICU hospitalizations in the general population, all hospitalizations, hospitalizations with admission to ICU, and the changes of incidence in each stratum from 2001 to 2010 are detailed in Table 1.

**Table 1:** Overall and Age-Stratified Annual Incidence of Candidemia-Associated Hospitalizations 18 Years or Older Admitted to ICU in Texas, 2001 - 2010

Incidence group	2001	2002	2003	2004	2005	2006	2007	2008	2009	2010	Change 2001 vs. 2010 (%)^a^
Population^b^											
All	2.73	3.08	3.42	3.47	5.16	5.22	5.26	5.1	5.47	5.22	91
18 - 44	0.67	1.07	1.13	1.1	1.36	1.49	1.38	1.36	1.5	1.41	110
45 - 64	3.14	3.28	3.94	3.96	6.31	5.61	6.43	6.12	6.99	6.13	96
65 - 84	10.17	10.68	11.69	12.19	17.59	19.79	17.48	17.06	17.69	16.19	59
≥ 85	12.19	13.84	12.83	11.4	22.53	16.44	22.3	21.83	17.83	23.92	96
Hospitalizations^c^											
All	1.61	1.79	1.99	2.06	3.09	3.14	3.21	3.2	3.47	3.34	107
18 - 44	0.77	1.21	1.2	1.28	1.62	1.76	1.64	1.67	1.85	1.72	123
45 - 64	2.72	2.55	3.33	3.41	5.49	4.91	5.69	5.55	6.3	5.59	106
65 - 84	3.03	3.17	3.46	3.66	5.34	6.13	5.57	5.61	6.01	5.72	82
≥ 85	2.09	2.09	2.12	1.95	3.79	2.83	3.89	3.79	3.21	4.32	107
ICU admissions^d^											
All	8.33	8.95	9.66	9.57	14.15	14.03	14.15	13.8	14.57	13.77	65
18 - 44	6.87	10.2	10.38	9.75	12.2	13.12	11.99	11.86	12.46	11.42	66
45 - 64	8.57	8.57	10.09	10.05	16.07	14.03	16.09	15.51	17.31	15.48	81
65 - 84	8.89	8.97	9.62	9.88	14.09	15.67	14.09	13.99	14.67	14.03	58
≥ 85	7.7	7.84	6.92	6.01	11.23	8	10.61	10.02	8.23	11.01	43

^a^Change of candidemia incidence from 2001 to 2010. ^b^State population, per 100,000. ^c^All hospitalizations, per 10,000. ^d^Hospitalizations with admission to ICU, per 10,000.

The incidence of C-ICU followed a biphasic pattern for all examined groups, generally rising rapidly during the first half of the decade, with subsequent relative plateau for the remainder of the decade.

The annual volume of C-ICU hospitalizations increased from 402 in 2001 to 954 in 2010, and the overall annual incidence of C-ICU in the general population rose by 91% at the end of the last decade, increasing between 2001 and 2010 from 2.73 to 5.22 hospitalizations per 100,000 population, respectively. When benchmarked for growth in the volume of all hospitalizations and those with ICU admission, the annual incidence of C-ICU rose by 107% and 65%, respectively. Although the incidence of C-ICU increased in all age strata, the relative changes for each over the decade varied substantially. Thus, the highest growth rate in the incidence of C-ICU among all hospitalizations (123%) occurred in the 18 - 44 year group.

The incidence of C-ICU increased with age, being highest in those 85 years or older in the general population, but peaked in the 45 - 84 year group, when benchmarked against all hospitalizations and those with ICU admission.

## Discussion

We found that by the end of the last decade, the overall and age-stratified annual incidence of C-ICU outpaced the growth in state’s population, all hospitalizations, and those with ICU admission, as compared to 2001. However, the observed changes in the incidence of C-ICU generally followed a biphasic course, with initial rapid rise followed by a relative plateau, thus reflecting more closely during the second half of the last decade the growth of state’s population and that of all hospitalizations and those with ICU admission.

The incidence of C-ICU in the general population rose rapidly with age, being over 16-fold higher among those aged ≥ 85 years, as compared to the 18 - 44 year group, with the difference maintained throughout study period, in line with prior reports [3, 5]. On the other hand, when benchmarked against all hospitalizations and those with ICU admission, the peak incidence was in the 45 - 84 year group, decreasing among those 85 years or older. Similar incidence differences across age groups were reported by other investigators [5] and may reflect distinct variation in background risk for candidemia, both in terms of illness and therapeutic interventions among hospitalized patients, as compared to the general population. However, the incidence of C-ICU was generally more closely clustered between the examined age groups when benchmarked against ICU admissions, as compared to that benchmarked against all hospitalizations. This difference probably reflects more comparable distribution of high-risk clinical conditions and level of exposure to external interventions across examined age groups in ICU populations.

Although there was marked rise in the incidence of C-ICU across all age groups over time, by the end of the last decade, there was substantial heterogeneity in its magnitude. Thus, the highest growth rate in the incidence of C-ICU was noted among the 18 - 44 year group both in the general population and when benchmarked against all hospitalizations. On the other hand, the elderly group aged 65 - 84 years showed the lowest rate of incidence rise in the same two categories. Finally, C-ICU among those 85 years or older had the lowest rate of incidence rise among all ICU admissions, being about half the rate of incidence growth observed in the 45 - 64 year age group. The observed heterogeneity in the pace of observed change in the incidence of C-ICU is likely related to a differential evolvement of burden of precipitating comorbidity and exposure to interventional risk (i.e., immunosuppressive therapy, invasive procedure) over time, and requires further study among patients admitted to ICU to better evaluate the sources of the observed patterns.

The observed relatively rapid rise in the incidence of C-ICU during the first half of the last decade is in line with national trends in the US among all candidemia hospitalizations reported by Zilberberg and colleagues [5] for the period of 2000 - 2005. There have been no longitudinal national or state-level reports, to our knowledge, on the incidence of candidemia in the US for the remainder of the last decade. However, a recent study by Cleveland et al [4], using active surveillance, demonstrated substantial decline in the incidence of candidemia in the metropolitan areas of Atlanta, Georgia and Baltimore, Maryland. In the only longitudinal population-level study to date focused on ICU patients in the US, the incidence of hospital-acquired candidemia decreased from 1989 to 1999 [12]. However, the findings of the latter report cannot be readily compared to the present study due to use of candidemia events benchmarked only against central venous catheter days [8].

Only limited data were reported on contemporary longitudinal patterns of the incidence of candidemia among ICU patients. The incidence of candidemia in the ICU was either unchanged (during 2006 - 2011) [8] or stable (during 2004 - 2006), with subsequent progressive rise (during 2006 - 2009) [9] in a national study [8] and an investigation of metropolitan areas [9] in Europe. The findings of the latter reports are not directly comparable to the present study due to their restriction to either primary nosocomial candidemia [8] or that developing when the patient was physically in the ICU [9].

The differences in the longitudinal direction of the incidence of C-ICU in the present study, as compared to prior ICU-focused reports are in line with the well-described substantial differences in the incidence of candidemia within [4] and between different nations [3, 6] reported in population-based studies not restricted to ICU populations. However, the sources for the observed variability remain unknown.

The driving factors underlying the relative plateau in the incidence of C-ICU across in the general population and following benchmarking against all hospitalizations and those with ICU admission during the second half of the last decade are uncertain. Improvement in clinical practice has been proposed as an explanation for the plateaued incidence of candidemia during the years 2001 - 2011 in Iceland [6]. A more recent surveillance-based report by Cleveland et al noted that after progressive rise, the incidence of candidemia dropped substantially between 2008 and 2013 in two metropolitan areas in the US [4]. The investigators hypothesized that the reversed incidence pattern is not likely due to changes in high-risk populations, but is probably related to improved healthcare delivery. They indicated that their hypothesis is supported by finding of unchanged incidence of community-acquired candidemia, but a decrease in that associated with healthcare exposure, and specifically decrease of candidemia among those with central venous catheters. However, the national study by Trick and colleagues, cited earlier, noted nearly three-fold drop in central line-associated candidemia per central line days [12], well before the recent demonstration of the effectiveness of central line care bundles. Pronovost [13] and previous reports indicated that the substantial decline in the rate of central line-associated BSI was less evident for those to candidemia [1, 13, 14]. Similarly, the hypothesis of Cleveland et al is difficult to reconcile with the diverging direction of the incidence of candidemia recently reported among ICU patients in Europe [8, 9].

Our findings should be considered in the context of several limitations. First, a retrospective design and use of an administrative data set with their attendant limitations affects the interpretation of our results. However, similar approach with the aforementioned limitations was used by other investigators [5, 7].

We used a specific ICD-9-CM code to identify candidemia hospitalizations and it is possible that some hospitalizations have been misclassified. The sensitivity and specificity of the code for disseminated candidiasis in identifying candidemia in administrative data sets is unknown. However, other investigators have used similar approach [5, 7], though it is possible that we underestimated the burden of candidemia [7]. Nevertheless, it is unlikely that widespread changes to in coding for candidemia account for the observed biphasic changes in the incidence of C-ICU and the differential age-specific changes over time in the present study.

We examined C-ICU in a large state with diverse population. However, the characteristics of C-ICU likely vary across states and nationally. Further studies on C-ICU are needed in other healthcare environments.

In conclusion, we demonstrated in the largest ICU-focused study to date a major, though age-variable, rise in the incidence of candidemia hospitalizations managed in the ICU over the past decade. However, there was a biphasic pattern across examined age strata with the incidence of candidemia plateauing over the second half of the decade, matching the corresponding growth in state’s population and that of all hospitalizations and those with admission to ICU. Further studies are warranted to corroborate our findings.
